# Magnetic Resonance Imaging Characteristics of Molecular Subgroups in Pediatric H3 K27M Mutant Diffuse Midline Glioma

**DOI:** 10.1007/s00062-021-01120-3

**Published:** 2021-12-17

**Authors:** Annika Hohm, Michael Karremann, Gerrit H. Gielen, Torsten Pietsch, Monika Warmuth-Metz, Lindsey A. Vandergrift, Brigitte Bison, Annika Stock, Marion Hoffmann, Mirko Pham, Christof M. Kramm, Johannes Nowak

**Affiliations:** 1 Neuroradiological Reference Center for the Pediatric Brain Tumor (HIT) Studies of the German Society of Pediatric Oncology and Hematology, Würzburg University Hospital, Würzburg, Germany; 2 Department of Neuroradiology, Würzburg University Hospital, Würzburg, Germany; 3 Current address: Division of Pediatric Stem Cell Transplantation and Immunology, University Children’s Medical Clinic, University Medical Center Hamburg-Eppendorf, Hamburg, Germany; 4 Department of Pediatric and Adolescent Medicine, University Medical Center Mannheim, Medical Faculty Mannheim, Heidelberg University, Mannheim, Germany; 5 Institute of Neuropathology, University Hospital Bonn, Bonn, Germany; 6 Departments of Radiology and Pathology, Massachusetts General Hospital, Harvard Medical School, Boston, MA USA; 7 Current address: Neuroradiological Reference Center for the Pediatric Brain Tumor (HIT) Studies of the German Society of Pediatric Oncology and Hematology, Department of Neuroradiology, University Augsburg, Faculty of Medicine, Augsburg, Germany; 8 Division of Pediatric Hematology and Oncology, University Medical Center Göttingen, Göttingen, Germany; 9 SRH Poliklinik Gera GmbH, Radiology Gotha, Gotha, Germany

**Keywords:** Pediatric brain tumors, Imaging, Molecular subgroups, Radiogenomics, WHO classification

## Abstract

**Purpose:**

Recent research identified histone H3 K27M mutations to be associated with a dismal prognosis in pediatric diffuse midline glioma (pDMG); however, data on detailed MRI characteristics with respect to H3 K27 mutation status and molecular subgroups (H3.1 and H3.3 K27M mutations) are limited.

**Methods:**

Standardized magnetic resonance imaging (MRI) parameters and epidemiologic data of 68 pDMG patients (age <18 years) were retrospectively reviewed and compared in a) H3 K27M mutant versus H3 K27 wildtype (WT) tumors and b) H3.1 versus H3.3 K27M mutant tumors.

**Results:**

Intracranial gliomas (*n* = 58) showed heterogeneous phenotypes with isointense to hyperintense signal in T2-weighted images and frequent contrast enhancement. Hemorrhage and necrosis may be present. Comparing H3 K27M mutant to WT tumors, there were significant differences in the following parameters: i) tumor localization (*p* = 0.001), ii) T2 signal intensity (*p* = 0.021), and iii) T1 signal homogeneity (*p* = 0.02). No significant imaging differences were found in any parameter between H3.1 and H3.3 K27M mutant tumors; however, H3.1 mutant tumors occurred at a younger age (*p* = 0.004). Considering spinal gliomas (*n* = 10) there were no significant imaging differences between the analyzed molecular groups.

**Conclusion:**

With this study, we are the first to provide detailed MR imaging data on H3 K27M mutant pDMG with respect to molecular subgroup status in a large patient cohort. Our findings may support diagnosis and future targeted therapeutic trials of pDMG within the framework of the radiogenomics concept.

**Supplementary Information:**

The online version of this article (10.1007/s00062-021-01120-3) contains supplementary material, which is available to authorized users.

## Introduction

Molecular characteristics of pediatric brain tumors have become an integral aspect of risk-adapted treatment strategies [1, 2]. Based on the revised classification of tumors of the central nervous system (CNS) by the World Health Organization (WHO) in 2016, the novel entity diffuse midline glioma, H3 K27M-mutant has been defined [3–5]. It includes the majority of diffuse high-grade gliomas (HGG) that emerge in midline structures of the CNS and typically affect children and young adults [5].

The leading molecular characteristic is a mutation of genes encoding histone H3 with replacement of a key lysine residue at position 27 by a methionine residue (K27M); this occurs in the *HIST1H3B/C* and *H3F3A *genes, among others, which encode for the variants H3.1 and H3.3, respectively [6–9]. The H3 K27M mutation is suggested to drive gliomagenesis, inhibit physiological cell differentiation [10–14], and maintain tumor growth [15]. The variants H3.1 and H3.3 K27M mutant tumors define distinct subgroups with differing prognosis and potentially phenotypes [16].

Although H3 K27M mutant diffuse midline gliomas (DMG) also occur in adults, differences in tumor biology of pediatric and adult tumors have been suggested [17–20]. In children particularly, H3 K27M mutations have been associated with infiltrative growth and a dismal prognosis, corresponding to WHO grade IV, regardless of tumor histology [11, 21, 22]; however, tumor biopsy is often challenging due to the location in midline CNS structures (mainly pons, thalamus, and spinal cord) [7, 13, 23]. To develop new therapeutic interventions that target the described histone mutations or underlying epigenetic pathways, a clinical/imaging surrogate marker of molecular subgroup status in DMG is desirable.

Only very limited data have been published using magnetic resonance imaging (MRI) to characterize H3 K27M mutant pediatric DMG (pDMG). Aboian et al. first described MRI features with variable contrast enhancement, edema, and necrosis but without significant differences from H3 K27 wildtype (WT) pDMG [24]. Another study by Rodriguez Gutierrez et al. in non-brainstem pediatric HGG further suggested a higher proportion of strongly enhancing H3 K27M mutant compared to WT tumors in the HERBY cohort [25]. In spinal pDMG, hemorrhage may be the only significantly discriminating feature between H3 K27M mutants and their WT counterparts in children and adults [26].

Some authors described MRI characteristics in H3 K27M subgroups (including H3.1 and H3.3 mutation) in diffuse intrinsic pontine glioma (DIPG) and found differences in the apparent diffusion coefficient (ADC) distribution and contrast enhancement patterns between subgroups [16, 27]; however, detailed data on MRI phenotypes of pDMG considering H3 K27 mutation status including molecular subgroups are still lacking.

In this study we describe and compare MRI of pDMG with known H3 K27 mutation status including H3.1 and H3.3 K27M subgroups in a large patient cohort. Radiological, pathological, and clinical data were retrieved from the HIT-HGG database of the Society of Pediatric Oncology and Hematology (Gesellschaft für Pädiatrische Onkologie und Hämatologie, GPOH). Our results may support diagnosis and future therapeutic trials of pDMG.

## Methods

### Patient Characteristics

A total of 85 pediatric patients with DMG were retrieved from the HIT-HGG registry (C.M.K., M.H., M.K.) of the GPOH. Patients were diagnosed between 1999 and 2016. Corresponding histopathological and molecular genetic data including H3 K27 mutation status were obtained from the German Neuropathological Reference Center for Pediatric Brain Tumor (HIT) Studies (T.P., G.H.G.). The MRI studies at diagnosis were obtained from the imaging database of the German Neuroradiological Reference Center for Pediatric Brain Tumor (HIT) Studies (M.W.-M., J.N., A.S.). Clinical and epidemiological data of the patient cohort have already been published [22].

Inclusion criteria of the presented study were patient age < 18 years at diagnosis, known molecular genetic H3 K27 mutation status (for *HIST1H3B/C *and* H3F3A*, resulting in H3.1 K27M or H3.3 K27M mutants, respectively, or H3 K27 WT), and availability of MRI scans at diagnosis with at least T1-weighted and T2-weighted images as well as T1-weighted images after gadolinium injection. Finally, MRI scans of 68 patients were analyzed.

The analysis of patient and MRI data was approved by the local and central ethics committees of the HIT studies and performed in accordance with the Declaration of Helsinki. Informed consent for data storage and statistical analyses was given by all patients and/or their parents at the time of registration and treatment within the HIT-HGG studies.

### Image Analysis

The MRI scans were acquired with MR scanners of different manufacturers operating at field strengths between 1.0 and 3.0 T. Retrospective imaging review was performed separately by two experienced radiologists (M.W‑M. and J.N.) together with a medical student (A.H.). In cases of initial differences between both readers, cases were further discussed in a consensus meeting.

For intracranial tumors the following MRI characteristics were analyzed: tumor localization (thalamus or basal ganglia, midbrain/tectum, pons, spinal cord, others), tumor volume (length in coronal, sagittal, and axial planes; approximated by a * b * c * 0.5 cm^3^), and characteristics of tumor margins (well-defined, i.e. more than 90% of the tumor margin/moderately well-defined, more than 50% and up to 90% of the tumor margin/ill-defined, 50% or less of the tumor margin). The T1 and T2 tumor signals were characterized by intensity (hyperintense/isointense/hypointense) in relation to normal cortex and by homogeneity (homogeneous/predominantly homogeneous/predominantly inhomogeneous/inhomogeneous). Further criteria included contrast enhancement with respect to intensity (none/mild/intermediate/strong), enhancement pattern (homogeneous/predominantly homogeneous/predominantly inhomogeneous/inhomogeneous), volume of the enhancing tumor part in relation to the whole tumor mass (< 25%, 25–50%, 50–75%, 75–100%), and the presence of ring enhancement. Furthermore, the presence of necrosis, peritumoral edema (including edema width in cm), and cysts (including cyst signal intensity compared to cerebrospinal fluid, CSF) were evaluated. Intratumoral necrosis was defined as intratumoral fluid accumulation with peripheral (ring) enhancement. In addition, multifocality of the tumor, CSF dissemination (M0 or M1, M2, according to Chang et al. [28]), intracranial metastases at diagnosis, and extent of hydrocephalus (none/low grade/intermediate/high grade, defined by HIT criteria) were assessed.

Additional criteria (when available within the MRI dataset) included cellularity within the tumor mass suggested by diffusion-weighted imaging (DWI; hyperintense/isointense/hypointense, compared to normal tissue) and ADC (diffusion restriction/no diffusion restriction). Presence of tumor hemorrhage was evaluated in all MRI and, if available, computed tomography (CT) sequences, including susceptibility weighted imaging (SWI) or T2*-weighted imaging (signal loss/no signal loss in SWI/T2*). Calcifications were assessed in CT scans (none/fine/gross), when available.

The MRI morphology of spinal tumors was characterized separately by assessing tumor expansion in relation to anatomic localization (cervical/cervicothoracic/thoracic/thoracolumbar), tumor length (in the sagittal plane in cm and in vertebral segment count, following recommendations of a previous study [26]), swelling of the cord (yes/no), gadolinium enhancement (yes/no/unclear), and presence of necrosis or hemorrhage.

### Statistical Analysis

We compared epidemiological and imaging data of a) H3.1 versus H3.3 K27M mutant tumors versus WT tumors and b) H3.1 versus H3.3 mutant tumors. Imaging features of spinal tumors were studied separately as tumor imaging characteristics are different from intracranial tumors. Statistical two-group comparisons were conducted using Fisher’s exact test of independence for parameters with two categories. For parameters with three or more categories, the two-sided χ^2^-test or Fisher-Freeman-Halton test was applied. Continuous parameters were analyzed using Mann-Whitney *U* tests. Statistical three-group comparisons were conducted in order to test for possible discrimination of all three genotypes (H3.3, H3.1, WT) by MRI, using the two-sided χ^2^-test or Fisher-Freeman-Halton test for categorial and Kruskal-Wallis test for continuous variables. *P*-values < 0.05 were considered statistically significant. For subsequent intergroup comparisons of H3.1 and H3.3 mutant pDMG, the significance level was adjusted to *p* < 0.017 after applying Bonferroni correction. Analysis was performed using IBM SPSS Statistics software, RRID:SCR_019096, versions 24–26 (IBM, Armonk, NY, USA).

## Results

### Patient Cohort and Molecular Status

The MRI studies of 68 pediatric patients with DMG were analyzed for this study (*n* = 58 intracranial, *n* = 10 spinal). A total of 17/85 patients were excluded due to missing or incomplete MRI data at diagnosis (*n* = 16) and age ≥ 18 years at diagnosis (*n* = 1). Molecular analysis revealed H3.1 K27M mutant (*n* = 6, 8.8%), H3.3 K27M mutant (*n* = 46, 67.6%), and WT tumors (*n* = 16, 23.5%). There was a slight difference in median manifestation age between H3 K27M mutant (10.2 years, range 1.25–17.75 years) and WT (9.6 years, range 0.33–16.17 years) tumors (*U* = 367.00, *z* = −0.71, *p* = 0.48); however, the median age at diagnosis differed significantly between H3.1 (3.9 years, range 1.25–13.25 years) and H3.3 (10.4 years, range 4.00–17.75 years) mutant tumors (*U* = 38.00, *z* = −2.87, *p* = 0.004), an effect also seen when comparing age between H3.1 K27M vs. H3.3 K27M vs. H3 K27 WT (*H*(2) = 6.84; *p* = 0.03). Sex was equally distributed with 51.5% males and 48.5% females across our patient cohort without any significant differences comparing H3 K27 mutation status (*p* = 0.78, Fisher’s exact test).

### Tumor Localization

**Fig. 1:**
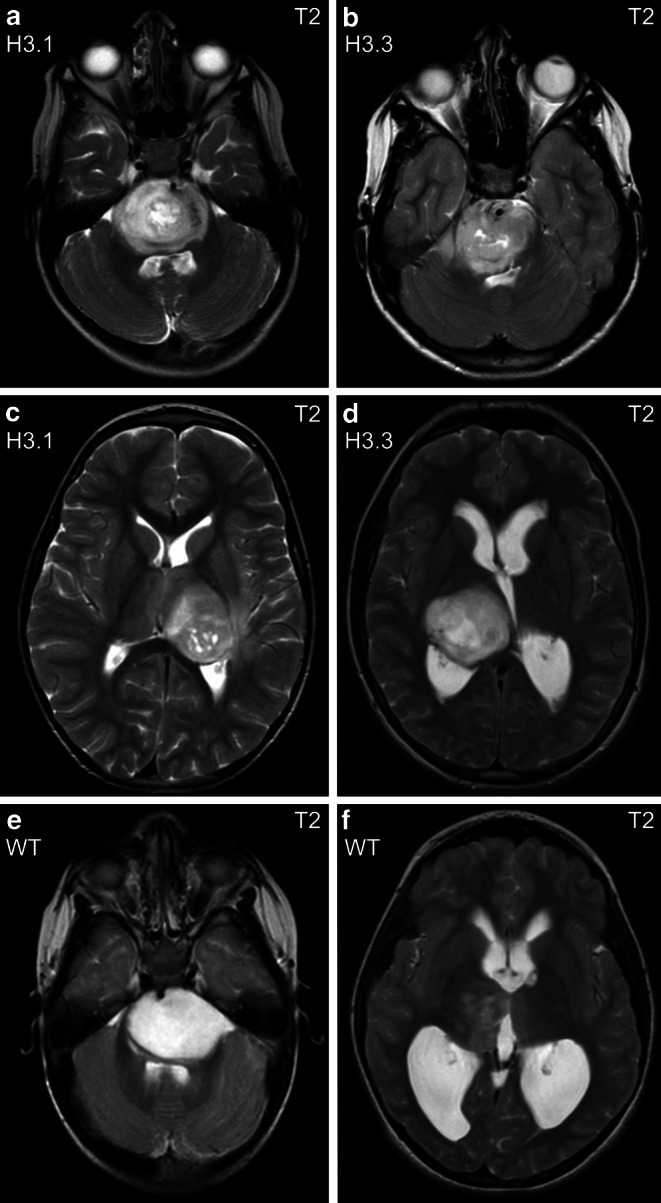
Typical localizations of pDMG in the pons (**a,** **b**) and thalamus (**c,** **d**) in axial MR images. Different H3 K27 genetic subgroups may show very similar MRI phenotypes with hyperintense, heterogeneous T2 signal (**a** and **b**, **c** and **d**). However, T2 signal may also appear very different even within the same genetic group (**e,** **f**)

**Fig. 2:**
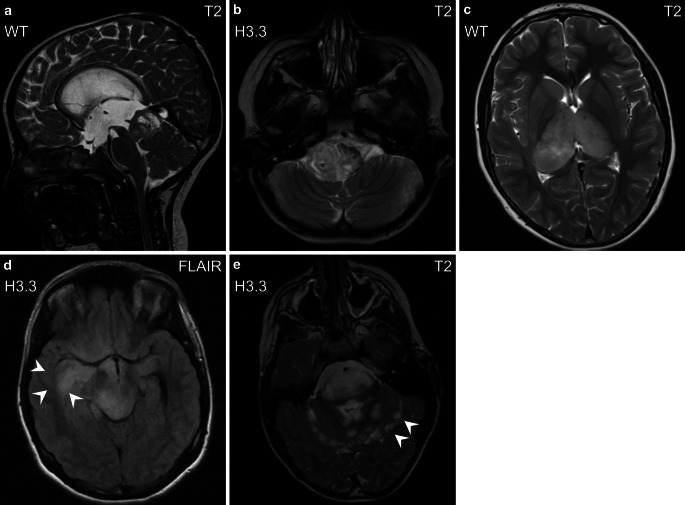
Other localizations of H3 K27M mutant and WT tumors in the midbrain/tectum (**a**, sagittal images) and medulla oblongata (**b**, axial images). Note that bithalamic gliomas (**c**, axial images) typically harbor epidermal growth factor receptor (EGFR) mutation rather than histone H3 mutation [29]. Rare MRI phenotypes with diffuse infiltrative growth (*arrowheads* in **d**, axial images) and with leptomeningeal spread at diagnosis (*arrowheads* in **e**, axial images)

The vast majority of H3 K27M mutants were found in the pons (51.9%) and thalamus/basal ganglia (36.5%) and further arose from spinal (9.6%) and other localizations (1.9%, including 1 tumor originating from the medulla oblongata). In contrast, H3 K27 WT tumors were more widely distributed (31.3% thalamus/basal ganglia, 31.3% spinal cord, 12.5% pons, 12.5% midbrain/tectum, 12.5% other intracranial localizations, including 1 tumor originating from the medulla oblongata and 1 tumor with relation to the lateral ventricle), thereby significantly differing from mutants (*p* = 0.001, Fisher-Freeman-Halton test). Tumor localization also differed significantly between H3.1 K27M vs. H3.3 K27M vs. H3 K27 WT (*p* = 0.01, Fisher-Freeman-Halton test) whereas molecular subgroups H3.1 and H3.3 did not differ (*p* = 1.00, Fisher-Freeman-Halton test). A detailed overview on tumor localization is given in Supplementary Table S1 (Online Resource 1). Tumors in typical localizations are exemplarily shown in Fig. 1. The variety of tumor distribution in pDMG is illustrated in Fig. 2.

### MR Imaging

#### H3 K27M versus H3 K27 WT

**Fig. 3:**
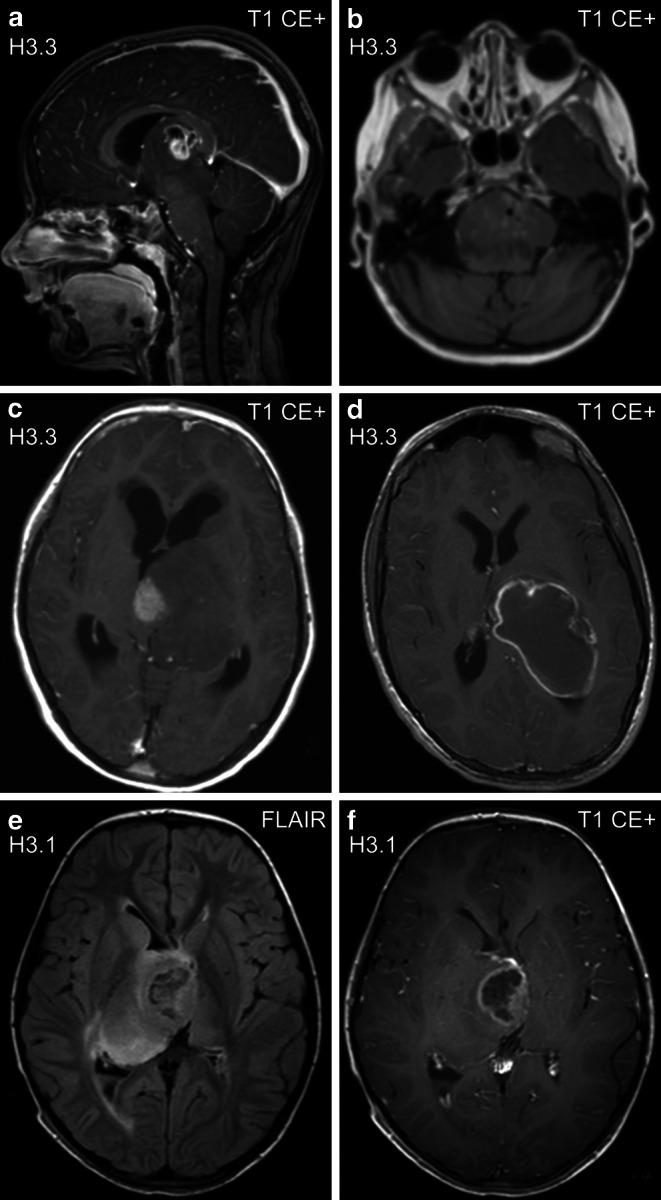
H3 K27M mutant tumors may show solid, enhancing, and necrotic portions (**a**, sagittal image)*, patchy enhancement (**b**, axial image), solid enhancing and non-enhancing areas (**c**, axial image), or necrosis only (**d**, axial image). Note that ring enhancement usually demarcates necrotic tumor portions (**e,** **f**; axial images). *Same patient as in Fig. 2a. *CE+* contrast enhanced

The majority of intracranial H3 K27M mutant tumors showed hyperintense T2 signal (93.6%), compared to only 63.6% in WT tumors (*p* = 0.02, Fisher’s exact test; remaining tumors: isointense). In T2-weighted images, H3 K27M mutant tumors appeared rather inhomogeneous (48.9% predominantly inhomogeneous, 8.5% inhomogeneous), whereas WT tumors displayed rather homogeneous T2 signal (63.6% predominantly homogeneous, 9.1% homogeneous) but not reaching significance (*p* = 0.06, Fisher-Freeman-Halton test). Fig. 1 shows selected T2-weighted MRI phenotypes. There was a statistically significant difference in T1 signal homogeneity, with H3 K27M mutants showing more inhomogeneous tumors (39.1% predominantly inhomogeneous, 2.2% inhomogeneous), compared to WT tumors (9.1% inhomogeneous, 0.0% predominantly inhomogeneous; *p* = 0.02, Fisher-Freeman-Halton test). Intratumoral hemosiderin deposits were more frequent in H3 K27M mutants (39.1%) than in WT tumors (9.1%, *p* = 0.08, Fisher-Freeman-Halton test). We did not see significant differences in any other parameter. Fig. 3 illustrates the spectrum of contrast enhancement in pDMG. Supplementary Table S2 (Online Resource 2) gives a detailed overview on all assessed imaging parameters.

#### H3.1 K27M versus H3.3 K27M

We did not find any MR imaging characteristic that differed significantly between H3.1 and H3.3 K27M mutant tumors. All assessed imaging parameters are displayed in Supplementary Table S2 (Online Resource 2). The MRI phenotypes of molecular subgroups are shown in Figs. 1 and 2.

#### H3.1 K27M versus H3.3 K27M versus H3 K27 WT

Testing for possible differentiation of all three genotypes (H3.1 K27M, H3.3 K27M, H3 K27 WT), we found significant differences regarding T2 signal intensity (*p* < 0.05, Fisher-Freeman-Halton test): H3.1 and H3.3 mutant tumors showed higher signal intensity (hyperintense: 100.0% and 92.7%, respectively) compared to WT tumors (hyperintense: 63.6%), hence complementing our findings with respect to H3 K27 mutation status (H3 K27M vs. H3 K27 WT). The MRI phenotypes are shown in Figs. 1 and 2, and additional imaging parameters and post hoc test information are listed in Supplementary Table S2 (Online Resource 2).

#### Spinal Tumors

**Fig. 4:**
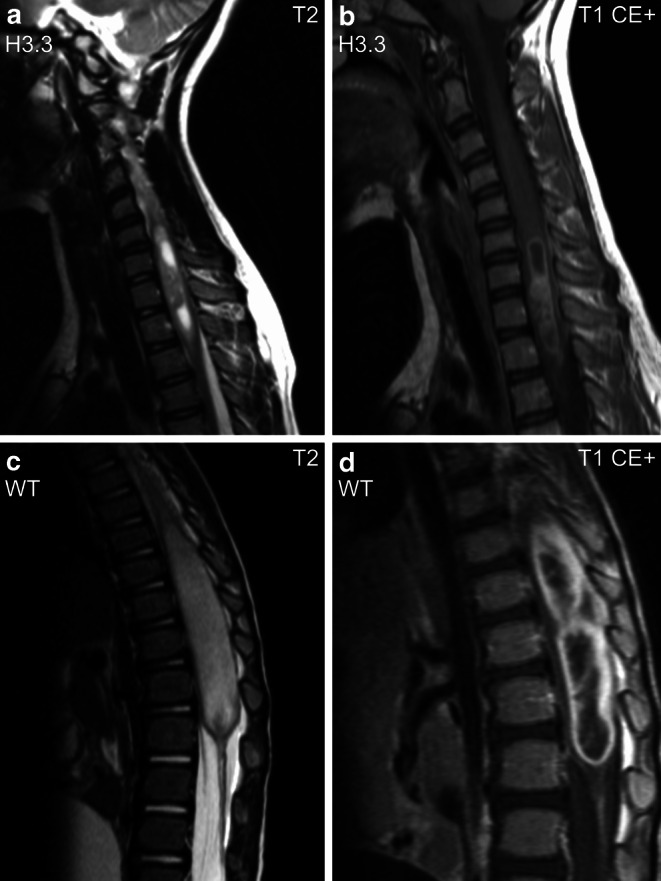
Spinal H3.3 K27M mutant tumor in the cervicothoracic region with cystoid necrosis and contrast enhancement (**a,** **b**; sagittal images). Spinal H3 K27 WT tumor with marked swelling of the conus medullaris and strong peripheral contrast enhancement (**c,** **d**; sagittal images). *CE+* contrast enhanced

Molecular analysis of spinal pDMG revealed H3.3 K27M mutant (*n* = 5) and WT tumors (*n* = 5). There were no spinal H3.1 K27M mutant tumors in our cohort. The majority of H3.3 mutant tumors originated in cervical (40.0%) and cervicothoracic segments (40.0%), thereby differing from WT cases which were located in lower spinal segments (60.0% thoracolumbar, 40.0% thoracic) but not reaching statistical significance (*p* = 0.06, Fisher-Freeman-Halton test). There were no imaging characteristics that significantly differentiated the two molecular groups. Imaging parameters of spinal pDMG are displayed in Supplementary Table S3 (Online Resource 3) and characteristic MR phenotypes are shown in Fig. 4.

## Discussion

With the genesis of the novel entity diffuse midline glioma, H3 K27M mutant in 2016 [3–5], various studies have been conducted to characterize and compare H3 K27M mutant tumors to their WT counterparts. In addition, biological and neuroanatomical differences between HGG of children and adults have been suggested [6, 7, 9, 15, 17, 18, 30, 31]. In our study, we, for the first time, systematically analyzed MR imaging features of 68 pDMG patients with known H3 K27 mutation status including H3.1 and H3.3 K27M molecular subgroups. We found that H3 K27M mutant and H3 K27 WT tumors may be differentiated by tumor localization, T2 signal intensity, and T1 signal homogeneity. The MRI phenotypes between H3.1 and H3.3 K27M subgroup tumors did not differ significantly, although differences in age of onset were identified. The clinical data of our pediatric cohort, including H3.1 and H3.3 subgroup-specific survival data, have already been published [22] and are now complemented by detailed MR imaging characteristics. The authors found that H3 K27M mutation was associated with shorter overall survival compared to H3 K27 WT and that H3 K27 mutation status was the only independent parameter to predict overall survival.

Regarding MR imaging in the pediatric population, few studies have aimed to characterize pDMG, DIPG, and non-brainstem HGG with respect to H3 K27 mutation status [16, 24, 25]. In a first study, Aboian et al. did not find any significant imaging differences between H3 K27M mutant pDMG and their WT counterparts [24]; however, data were based on a relatively small cohort, and there was no differentiation of mutant tumors into H3.1 and H3.3 mutant subgroups. Regarding H3.1 and H3.3 K27M subgroups in DIPG, Castel et al. suggested that H3.1 subgroup tumors show distinct contrast enhancement patterns (more frequent tumors with large necrotic areas and ring or nodular enhancement) and present lower ADC values [16]. H3.1 mutant patients presented at a younger age, had better clinical treatment response and fewer metastatic recurrences compared to patients with H3.3 K27M subgroup DIPG. In contrast, in a recent study H3.1 K27M subgroup DIPG presented higher ADC values [27]. A radiological evaluation of pediatric non-brainstem HGG within the HERBY trial revealed increased contrast enhancement in H3 K27M mutant tumors, not differentiating between H3.1 and H3.3 subgroups [25]; however, a study on pDMG with and without H3 K27M mutations did not find differences regarding the presence of contrast enhancement and necrotic areas with respect to mutation status [32]. Furthermore, a recent study performed by Thust et al. in a relatively small cohort of H3 K27M mutant pediatric and young adult DMG (*n* = 15) did not find characteristic MR morphologic parameters including diffusivity for this tumor entity; however, a control group of WT tumors was absent [33].

Using quantitative imaging techniques, no differences were found in ADC histogram analysis comparing H3 K27M mutant or WT pDMG but H3.1 and H3.3 K27M subgroups were not addressed [34]. A study applying advanced MR imaging revealed H3 K27M mutant tumors to exhibit higher relative arterial spin labeling-derived maximum cerebral blood flows, lower DWI-derived relative minimum ADC values and higher choline to N‑acetyl aspartate peak area ratios in ^1^H‑MR spectroscopy. In positron emission tomography (PET) analysis, higher ^18^F‑dihydroxyphenylalanine (DOPA) uptake was found in H3 K27M mutants compared to WT tumors [32].

In our study, we confirmed the typical localization of H3 K27M mutant pDMG, as previously reported [5, 23]: 88.5% of H3 K27M mutant tumors arose in a pontine or thalamic and 9.6% in a spinal localization, whereas H3 K27 WT tumors were more widely distributed (*see* Supplementary Table S1, Online Resource 1, and also detailed clinical data of our cohort as published by Karremann et al. [22]). We first reported significant differences between H3 K27M mutant tumors and their WT counterparts in terms of T2 signal intensity and T1 signal homogeneity. From a radiological perspective, higher T2 signals may point to lower cellularity (in H3 K27M mutant tumors) and in MR imaging of brain tumors there is a trend to inhomogeneous signal (more frequently seen in H3 K27M mutant pDMG) being linked with more aggressive growth; however, these findings are also subject to interobserver bias and as we did not find correlating differences in other standard MR sequences (such as DWI and contrast-enhanced T1), our findings alone may not differentiate H3 K27M mutant from WT tumors. Interestingly, Jansen et al. linked ring enhancement in DIPG (not addressing mutation status at that time) in MRI with a shorter overall survival [35]. For pDMG, a worse clinical course and shorter survival was hence linked to an H3 K27M mutant status [22]. In our imaging cohort, ring enhancement was exhibited more frequently by H3 K27M mutant (53.2%) compared to H3 K27 WT (36.4%) tumors but this finding did not reach statistical significance.

We are the first to describe that MRI phenotypes of H3.1 and H3.3 K27M subgroups of pDMG may not differ but we do see that H3.1 subgroup pDMG occurred at a significantly younger age. This finding is in line with previously described findings in DIPG and pDMG [16, 22], pointing to differences in tumor biology between the subgroups. Interestingly, a single case of our cohort presenting with metastases at diagnosis carried an H3.3 K27M mutation, potentially supporting previous observations of a higher tendency towards metastatic relapse in H3.3 K27M mutant DIPG [16].

Few studies show single MRI cases of spinal H3 K27M mutant DMG in different age groups, not differentiating between H3.1 and H3.3 subgroups [24, 31, 36–39]. With our study, we, for the first time, systematically analyzed MRI parameters of spinal DMG in the pediatric population with respect to H3 K27 mutation status but there was no H3.1 mutant spinal tumor in our cohort. In a study by Jung et al. in pediatric and adult spinal DMG, hemorrhage was the only imaging parameter that differed significantly (more frequently in H3 K27M mutants compared to no hemorrhage in WT tumors) [26]. In our study, H3.3 K27M and WT pDMG did not differ significantly regarding the presence of hemorrhage, with a single WT case showing clear signs of hemorrhage; however, the localization differed slightly, with H3.3 K27M pDMG arising in higher spinal segments than WT tumors but not reaching significance (*p* = 0.06). In summary, a differentiation of molecular status in spinal pDMG by means of MRI may not be feasible at present.

This study is limited by its retrospective design and heterogeneous MRI data provided by the referring centers within the HIT trials. Therefore, quantitative analysis of MR parameters (such as diffusivity) or analysis of advanced imaging techniques (such as MR perfusion or MR spectroscopy) and machine learning-based approaches could not be investigated comparatively. Only a few studies applying machine learning-based models to predict H3 K27M mutation in spinal glioma, brainstem glioma, or midline glioma based on conventional MRI parameters have been published [26, 40–42]. Furthermore, reliability of data evaluation based on consensus reading of two experienced experts was not statistically validated and patients of our cohort were not evenly distributed along the assessed molecular groups, impeding statistical analysis.

Nevertheless, the multicenter design (referring to a network of reference centers for oncology, neuropathology, and neuroradiology) provides a comparatively large cohort of pDMG cases with known H3 K27 mutation status, including a WT control group.

## Conclusion

A differentiation of molecular status in diffuse midline glioma, H3 K27M mutant using conventional MRI seems challenging at present. We are the first to report that this may also apply for H3.1 and H3.3 K27M subgroups in children, as well as for their discrimination from WT tumors. Further investigations applying machine learning-based approaches (implementing imaging, clinical, and molecular data) are needed to design a non-invasive prediction model for H3 K27 mutation status of these rare tumors with a dismal prognosis.

## Supplementary Information

Supplementary Table S1

Supplementary Table S2

Supplementary Table S3
