# Supplementary material for: Magnetic Resonance Imaging Characteristics of Molecular Subgroups in Pediatric H3 K27M Mutant Diffuse Midline Glioma
Source: Clin Neuroradiol. 2021 Dec 17;32(1):249–58. doi: 10.1007/s00062-021-01120-3 (PMC8894220; doi:10.1007/s00062-021-01120-3)
Supplement: Supplementary file 1 — Supplementary Table S1 [file 62_2021_1120_MOESM1_ESM.pdf]

Supplementary Information – Online Resource 1

Article: Magnetic resonance imaging characteristics of molecular subgroups in pediatric H3 K27M mutant diffuse midline glioma

Journal: Clinical Neuroradiology

Authors: Annika Hohm[1,5], Michael Karremann[2], Gerrit H. Gielen[3], Torsten Pietsch[3], Monika Warmuth-Metz[1,5], Lindsey A. Vandergrift[4], Brigitte Bison[1], Annika Stock[1,5], Marion Hoffmann[6], Mirko Pham[5], *Christof M. Kramm[6], *Johannes Nowak[1,5,7,‡]

1 Neuroradiological Reference Center for the pediatric brain tumor (HIT) studies of the German Society of Pediatric Oncology and Hematology, Würzburg University Hospital (until 2020), Department of Neuroradiology, University Augsburg, Faculty of Medicine (since 2021), Germany

2 Department of Pediatric and Adolescent Medicine, University Medical Center Mannheim, Medical Faculty Mannheim, Heidelberg University, Mannheim, Germany

3 Institute of Neuropathology, University Hospital Bonn, Bonn, Germany

4 Departments of Radiology and Pathology, Massachusetts General Hospital, Harvard Medical School, Charlestown, Massachusetts, USA

5 Department of Neuroradiology, Würzburg University Hospital, Würzburg, Germany

6 Division of Pediatric Hematology and Oncology, University Medical Center Göttingen, Göttingen, Germany

7 SRH Poliklinik Gera GmbH, Radiological Practice Gotha, Gotha, Germany

*These authors contributed equally to this work.

[‡]Corresponding author: Johannes Nowak (Johannes.Nowak@yahoo.de), primary affiliation: Department of Neuroradiology, Würzburg University Hospital, Würzburg, Germany

Supplementary Table S1
Epidemiological data and tumor characteristics (grading, localization) of H3 (H3.1/H3.3) K27M mutant and H3 K27 WT pDMG (absolute and relative (%) frequencies are displayed). Statistically significant differences (*p*<.05; for H3.1 vs. H3.3, H3.1 vs. WT, and H3.3 vs. WT *p*<.017) are shown in boldface.

	Total pDMG (*n* = 68)	H3 K27M (*n* = 52)	H3 K27M subgroups		H3 K27 WT (*n* = 16)	*p*-value				
			H3.1 K27M (*n* = 6)	H3.3 K27M (*n* = 46)		H3 K27M vs. WT	H3.1 vs. H3.3	H3.1 vs. WT	H3.3 vs. WT	H3.1 vs. H3.3 vs. WT
Age (years), median [IQR]	10.2 [6.3-13.1]	10.2 [6.9-13.0]	3.9 [3.1-6.8]	10.4 [7.7-13.1]	9.6 [1.5-13.6]	.48[a]	**.004**[a]	.51[a]	.35[a]	**.03**[b]
Sex, *n* (%)						.78[c]	n.a.[d]	1.00[c]	.78[c]	.93[e]
Female	33 (48.5)	26 (50.0)	3 (50.0)	23 (50.0)	7 (43.8)					
Male	35 (51.5)	26 (50.0)	3 (50.0)	23 (50.0)	9 (56.3)					
Grade, *n* (%)						.41[e]	.35[e]	.38[e]	.49[e]	.34[e]
WHO II	3 (4.4)	3 (5.8)	1 (16.7)	2 (4.3)	0 (0.0)					
WHO III	33 (48.5)	23 (44.3)	3 (50.0)	20 (43.5)	10 (62.5)					
WHO IV	32 (47.1)	26 (50.0)	2 (33.3)	24 (52.2)	6 (37.5)					
Anatomic localization, *n* (%)						**.001**[e]	1.00[e]	.13[e]	**.002**[e]	**.01**[e]
Thalamus/basal ganglia	24 (35.3)	19 (36.5)	2 (33.3)	17 (37.0)	5 (31.3)					
Midbrain tectum	2 (2.9)	0 (0.0)	0 (0.0)	0 (0.0)	2 (12.5)					
Pons	29 (42.6)	27 (51.9)	4 (66.7)	23 (50.0)	2 (12.5)					

Spinal cord	10 (14.7)	5 (9.6)	0 (0.0)	5 (10.9)	5 (31.3)
Other	3 (4.4)	1 (1.9)	0 (0.0)	1 (2.2)	2 (12.5)

Note. WT wildtype, pDMG pediatric diffuse midline glioma, IQR interquartile range, WHO World Health Organization. Relative frequencies summing up to 100% per column.
[a] Mann-Whitney *U* test. [b] Kruskal-Wallis test. [c] Fisher's exact test of independence. [d] No statistics performed as values are identical in both groups.
[e] Fisher-Freeman-Halton test.
