# Supplementary material for: Magnetic Resonance Imaging Characteristics of Molecular Subgroups in Pediatric H3 K27M Mutant Diffuse Midline Glioma
Source: Clin Neuroradiol. 2021 Dec 17;32(1):249–58. doi: 10.1007/s00062-021-01120-3 (PMC8894220; doi:10.1007/s00062-021-01120-3)
Supplement: Supplementary file 2 — Supplementary Table S2 [file 62_2021_1120_MOESM2_ESM.pdf]

Supplementary Information – Online Resource 2

Article: Magnetic resonance imaging characteristics of molecular subgroups in pediatric H3 K27M mutant diffuse midline glioma

Journal: Clinical Neuroradiology

Authors: Annika Hohm[1,5], Michael Karremann[2], Gerrit H. Gielen[3], Torsten Pietsch[3], Monika Warmuth-Metz[1,5], Lindsey A. Vandergrift[4], Brigitte Bison[1], Annika Stock[1,5], Marion Hoffmann[6], Mirko Pham[5], *Christof M. Kramm[6], *Johannes Nowak[1,5,7,‡]

1 Neuroradiological Reference Center for the pediatric brain tumor (HIT) studies of the German Society of Pediatric Oncology and Hematology, Würzburg University Hospital (until 2020), Department of Neuroradiology, University Augsburg, Faculty of Medicine (since 2021), Germany

2 Department of Pediatric and Adolescent Medicine, University Medical Center Mannheim, Medical Faculty Mannheim, Heidelberg University, Mannheim, Germany

3 Institute of Neuropathology, University Hospital Bonn, Bonn, Germany

4 Departments of Radiology and Pathology, Massachusetts General Hospital, Harvard Medical School, Charlestown, Massachusetts, USA

5 Department of Neuroradiology, Würzburg University Hospital, Würzburg, Germany

6 Division of Pediatric Hematology and Oncology, University Medical Center Göttingen, Göttingen, Germany

7 SRH Poliklinik Gera GmbH, Radiological Practice Gotha, Gotha, Germany

*These authors contributed equally to this work.

[‡]Corresponding author: Johannes Nowak (Johannes.Nowak@yahoo.de), primary affiliation: Department of Neuroradiology, Würzburg University Hospital, Würzburg, Germany

Supplementary Table S2
MR imaging characteristics of intracranial H3 (H3.1/H3.3) K27M mutant and H3 K27 WT pDMG (absolute and relative (%) frequencies are displayed). All assessed MRI parameters are displayed. Statistically significant differences (p<.05; for H3.1 vs. H3.3, H3.1 vs. WT, and H3.3 vs. WT p<.017) are shown in boldface.

	Intracranial pDMG (n = 58)	H3 K27M (n = 47)	H3 K27M subgroups		H3 K27 WT (n = 11)	p-value				
			H3.1 K27M (n = 6)	H3.3 K27M (n = 41)		H3 K27M vs. WT	H3.1 vs. H3.3	H3.1 vs. WT	H3.3 vs. WT	H3.1 vs. H3.3 vs. WT
Tumor volume in cm³, median [IQR][a]	34.26 [22.60-42.73]	35.15 [23.60-43.39]	31.51 [22.83-45.85]	35.41 [23.48-44.22]	25.38 [9.79-49.83]	.11[b]	.73[b]	.33[b]	.11[b]	.26[c]
Tumor margins, n (%)	58	47	6	41	11	.44[d]	1.00[d]	.60[e]	.38[d]	.74[d]
Well-defined	3 (5.2)	3 (6.4)	0 (0.0)	3 (7.3)	0 (0.0)					
Moderately well-defined	31 (53.4)	23 (48.9)	3 (50.0)	20 (48.8)	8 (72.7)					
Ill-defined	24 (41.4)	21 (44.7)	3 (50.0)	18 (43.9)	3 (27.3)					
T1 tumor signal, n (%)	57	46	6	40	11	.18[e]	.39[e]	1.00[e]	.10[e]	.13[d]
Hyperintense	0 (0.0)	0 (0.0)	0 (0.0)	0 (0.0)	0 (0.0)					
Isointense	29 (50.9)	21 (45.7)	4 (66.7)	17 (42.5)	8 (72.7)					
Hypointense	28 (49.1)	25 (54.3)	2 (33.3)	23 (57.5)	3 (27.3)					
T1 tumor homogeneity, n (%)	57	46	6	40	11	**.02**[d]	.85[d]	.21[d]	.03[d]	.10[d]
Homogeneous	5 (8.8)	4 (8.7)	0 (0.0)	4 (10.0)	1 (9.1)					
Predominantly homogeneous	32 (56.1)	23 (50.0)	4 (66.7)	19 (47.5)	9 (81.8)					

Predominantly inhomogeneous	18 (31.6)	18 (39.1)	2 (33.3)	16 (40.0)	0 (0.0)					
Inhomogeneous	2 (3.5)	1 (2.2)	0 (0.0)	1 (2.5)	1 (9.1)					
T2 tumor signal, *n* (%)	58	47	6	41	11	**.02**[e]	1.00[e]	.24[e]	.03[e]	< .05[d]
Hyperintense	51 (87.9)	44 (93.6)	6 (100.0)	38 (92.7)	7 (63.6)					
Isointense	7 (12.1)	3 (6.4)	0 (0.0)	3 (7.3)	4 (36.4)					
Hypointense	0 (0.0)	0 (0.0)	0 (0.0)	0 (0.0)	0 (0.0)					
T2 tumor homogeneity, *n* (%)	58	47	6	41	11	.06[d]	1.00[d]	.11[d]	.09[d]	.21[d]
Homogeneous	4 (6.9)	3 (6.4)	0 (0.0)	3 (7.3)	1 (9.1)					
Predominantly homogeneous	24 (41.4)	17 (36.2)	2 (33.3)	15 (36.6)	7 (63.6)					
Predominantly inhomogeneous	24 (41.4)	23 (48.9)	4 (66.7)	19 (46.3)	1 (9.1)					
Inhomogeneous	6 (10.3)	4 (8.5)	0 (0.0)	4 (9.8)	2 (18.2)					
Contrast enhancement, *n* (%)										
• *Intensity*	58	47	6	41	11	.48[d]	.64[d]	.64[d]	.51[d]	.64[d]
Strong	21 (36.2)	19 (40.4)	2 (33.3)	17 (41.5)	2 (18.2)					
Intermediate	11 (19.0)	9 (19.1)	2 (33.3)	7 (17.1)	2 (18.2)					
Mild	10 (17.2)	7 (14.9)	0 (0.0)	7 (17.1)	3 (27.3)					
No enhancement	16 (27.6)	12 (25.5)	2 (33.3)	10 (24.4)	4 (36.4)					
• *Enhancement pattern (if existent)*	42	35	4	31	7	.20[d]	.80[d]	.82[d]	.32[d]	.44[d]
Homogeneous	1 (2.4)	0 (0.0)	0 (0.0)	0 (0.0)	1 (14.3)					

Predominantly homogeneous	9 (21.4)	7 (20.0)	0 (0.0)	7 (22.6)	2 (28.6)					
Predominantly inhomogeneous	20 (47.6)	18 (51.4)	3 (75.0)	15 (48.4)	2 (28.6)					
Inhomogeneous	12 (28.6)	10 (28.6)	1 (25.0)	9 (29.0)	2 (28.6)					
• *Volume of the enhancing part in relation to total tumor volume (if existent)*	42	35	4	31	7	1.00[d]	.79[d]	1.00[d]	1.00[d]	.97[d]
(0, 25] %	27 (64.3)	22 (62.9)	2 (50.0)	20 (64.5)	5 (71.4)					
(25, 50] %	6 (14.3)	5 (14.3)	1 (25.0)	4 (12.9)	1 (14.3)					
(50, 75] %	8 (19.0)	7 (20.0)	1 (25.0)	6 (19.4)	1 (14.3)					
(75, 100] %	1 (2.4)	1 (2.9)	0 (0.0)	1 (3.2)	0 (0.0)					
Ring enhancement, *n* (%)	58	47	6	41	11	.50[e]	.67[e]	.34[e]	.50[e]	.49[d]
Yes	29 (50.0)	25 (53.2)	4 (66.7)	21 (51.2)	4 (36.4)					
No	29 (50.0)	22 (46.8)	2 (33.3)	20 (48.8)	7 (63.6)					
Necrosis, *n* (%)	58	47	6	41	11	.32[e]	1.00[e]	.34[e]	.32[e]	.45[d]
Yes	31 (53.4)	27 (57.4)	4 (66.7)	23 (56.1)	4 (36.4)					
No	27 (46.6)	20 (42.6)	2 (33.3)	18 (43.9)	7 (63.6)					
Edema, *n* (%)	51	41	6	35	10	.67[e]	.58[e]	.52[e]	1.00[e]	.62[d]
Yes	9 (17.6)	8 (19.5)	2 (33.3)	6 (17.1)	1 (10.0)					
No	42 (82.4)	33 (80.5)	4 (66.7)	29 (82.9)	9 (90.0)					
Edema width in cm, median [IQR] *(if existent)*	0.44 [0.40-1.17]	0.41 [0.40-0.79]	0.63 [0.40-0.85]	0.41 [0.37-0.63]	1.41 [n.a.]	.12[b]	.56[b]	.22[b]	.14[b]	.27[c]
Cysts, *n* (%)	58	47	6	41	11					

• *Existence*						.16[e]	.24[e]	1.00[e]	.11[e]	.10[d]
Yes	4 (6.9)	2 (4.3)	1 (16.7)	1 (2.4)	2 (18.2)					
No	54 (93.1)	45 (95.7)	5 (83.3)	40 (97.6)	9 (81.8)					
• *Signal Intensity (if existent)*	4	2	1	1	2	1.00[e]	1.00[e]	.33[e]	n.a.[f]	.50[d]
Hyperintense related to CSF	3 (75.0)	1 (50.0)	0 (0.0)	1 (100.0)	2 (100.0)					
Isointense related to CSF	1 (25.0)	1 (50.0)	1 (100.0)	0 (0.0)	0 (0.0)					
CSF dissemination, *n* (%)	58	47	6	41	11	1.00[e]	.24[e]	.35[e]	1.00[e]	.23[d]
M2 (visible in MRI)	2 (3.4)	2 (4.3)	1 (16.7)	1 (2.4)	0 (0.0)					
M0 or M1	56 (96.6)	45 (95.7)	5 (83.3)	40 (97.6)	11 (100.0)					
Multifocality, *n* (%)	58	47	6	41	11	.47[e]	1.00[e]	1.00[e]	.52[e]	.66[d]
Yes	3 (5.2)	2 (4.3)	0 (0.0)	2 (4.9)	1 (9.1)					
No	55 (94.8)	45 (95.7)	6 (100.0)	39 (95.1)	10 (90.9)					
Metastases at diagnosis, *n* (%)	58	47	6	41	11	1.00[e]	1.00[e]	n.a.[f]	1.00[e]	1.00[d]
Yes	1 (1.7)	1 (2.1)	0 (0.0)	1 (2.4)	0 (0.0)					
No	57 (98.3)	46 (97.9)	6 (100.0)	40 (97.6)	11 (100.0)					
Hydrocephalus, *n* (%)	58	47	6	41	11	.21[d]	.50[d]	.06[d]	.39[d]	.25[d]
None	34 (58.6)	29 (61.7)	5 (83.3)	24 (58.5)	5 (45.5)					
Grade I	6 (10.3)	6 (12.8)	1 (16.7)	5 (12.2)	0 (0.0)					
Grade II	17 (29.3)	11 (23.4)	0 (0.0)	11 (26.8)	6 (54.5)					
Grade III	1 (1.7)	1 (2.1)	0 (0.0)	1 (2.4)	0 (0.0)					

DWI, *n* (%)	33	27	2	25	6	.44[d]	1.00[d]	1.00[e]	.47[d]	.65[d]
Hyperintense	27 (81.8)	22 (81.5)	2 (100.0)	20 (80.0)	5 (83.3)					
Isointense	4 (12.1)	4 (14.8)	0 (0.0)	4 (16.0)	0 (0.0)					
Hypointense	2 (6.1)	1 (3.7)	0 (0.0)	1 (4.0)	1 (16.7)					
ADC, *n* (%)	23	17	2	15	6	.16[e]	.52[e]	1.00[e]	.15[e]	.22[d]
Diffusion restricted	9 (39.1)	5 (29.4)	1 (50.0)	4 (26.7)	4 (66.7)					
No restriction	14 (60.9)	12 (70.6)	1 (50.0)	11 (73.3)	2 (33.3)					
SWI or T2*, *n* (%)	15	12	3	9	3	.20[e]	.52[e]	1.00[e]	.18[e]	.24[d]
Signal loss	7 (46.7)	7 (58.3)	1 (33.3)	6 (66.7)	0 (0.0)					
No signal loss	8 (53.3)	5 (41.7)	2 (66.7)	3 (33.3)	3 (100.0)					
Calcifications in CT, *n* (%)	17	13	3	10	4	.12[e]	1.00[e]	.43[e]	.18[e]	.18[d]
Gross calcifications	3 (17.6)	1 (7.7)	0 (0.0)	1 (10.0)	2 (50.0)					
Fine calcifications	0 (0.0)	0 (0.0)	0 (0.0)	0 (0.0)	0 (0.0)					
None	14 (82.4)	12 (92.3)	3 (100.0)	9 (90.0)	2 (50.0)					
Hemorrhage, *n* (%)	57	46	6	40	11	.08[e]	1.00[e]	.52[e]	.08[e]	.16[d]
Yes	19 (33.3)	18 (39.1)	2 (33.3)	16 (40.0)	1 (9.1)					
No	38 (66.7)	28 (60.9)	4 (66.7)	24 (60.0)	10 (90.9)					

Note. WT wildtype, pDMG pediatric diffuse midline glioma, IQR interquartile range, n.a. not applicable, CSF cerebrospinal fluid, DWI diffusion-weighted imaging, ADC apparent diffusion coefficient, SWI susceptibility-weighted imaging, CT computed tomography. Relative frequencies summing up to 100% per column.

[a] One H3 K27 WT case with diffuse tumor infiltration missing. [b] Mann-Whitney *U* test. [c] Kruskal-Wallis test. [d] Fisher-Freeman-Halton test. [e] Fisher's exact test of independence. [f] No statistics performed as values are identical in both groups.
