# Supplementary material for: Magnetic Resonance Imaging Characteristics of Molecular Subgroups in Pediatric H3 K27M Mutant Diffuse Midline Glioma
Source: Clin Neuroradiol. 2021 Dec 17;32(1):249–58. doi: 10.1007/s00062-021-01120-3 (PMC8894220; doi:10.1007/s00062-021-01120-3)
Supplement: Supplementary file 3 — Supplementary Table S3 [file 62_2021_1120_MOESM3_ESM.pdf]

Supplementary Information – Online Resource 3

Article: Magnetic resonance imaging characteristics of molecular subgroups in pediatric H3 K27M mutant diffuse midline glioma

Journal: Clinical Neuroradiology

Authors: Annika Hohm[1,5], Michael Karremann[2], Gerrit H. Gielen[3], Torsten Pietsch[3], Monika Warmuth-Metz[1,5], Lindsey A. Vandergrift[4], Brigitte Bison[1], Annika Stock[1,5], Marion Hoffmann[6], Mirko Pham[5], *Christof M. Kramm[6], *Johannes Nowak[1,5,7,‡]

1 Neuroradiological Reference Center for the pediatric brain tumor (HIT) studies of the German Society of Pediatric Oncology and Hematology, Würzburg University Hospital (until 2020), Department of Neuroradiology, University Augsburg, Faculty of Medicine (since 2021), Germany

2 Department of Pediatric and Adolescent Medicine, University Medical Center Mannheim, Medical Faculty Mannheim, Heidelberg University, Mannheim, Germany

3 Institute of Neuropathology, University Hospital Bonn, Bonn, Germany

4 Departments of Radiology and Pathology, Massachusetts General Hospital, Harvard Medical School, Charlestown, Massachusetts, USA

5 Department of Neuroradiology, Würzburg University Hospital, Würzburg, Germany

6 Division of Pediatric Hematology and Oncology, University Medical Center Göttingen, Göttingen, Germany

7 SRH Poliklinik Gera GmbH, Radiological Practice Gotha, Gotha, Germany

*These authors contributed equally to this work.

[‡]Corresponding author: Johannes Nowak (Johannes.Nowak@yahoo.de), primary affiliation: Department of Neuroradiology, Würzburg University Hospital, Würzburg, Germany

Supplementary Table S3
MR imaging characteristics of spinal H3.3 K27M mutant and H3 K27 WT pDMG (absolute and relative (%) frequencies are displayed). There were no spinal H3.1 K27M mutant cases in our cohort.

	Spinal pDMG (*n* = 10)	H3.3 K27M (*n* = 5)	H3 K27 WT (*n* = 5)	*p*-value H3.3 K27M vs. H3 K27 WT
Tumor localization (related to vertebral body)		C2 - C7<br>C3 - C7<br>C4 - T1<br>C6 - T2<br>T9 - T12	T2 - T5<br>T10 - T12<br>T6 - L3<br>T8 - L1<br>T9 - L2	n.a.
Anatomic localization, *n* (%)				.06[a]
Cervical	2 (20.0)	2 (40.0)	0 (0.0)	
Cervicothoracic	2 (20.0)	2 (40.0)	0 (0.0)	
Thoracic	3 (30.0)	1 (20.0)	2 (40.0)	
Thoracolumbar	3 (30.0)	0 (0.0)	3 (60.0)	
Tumor length in cm, median [IQR]	7.4 [5.3-8.2]	7.4 [5.3-8.3]	7.4 [5.2-10.1]	.92[b]
Involved vertebral segments, median [IQR]	5.0 [4.0-6.0]	5.0 [4.0-5.5]	6.0 [3.5-8.0]	.59[b]
Swelling, *n* (%)				n.a.[c]
Yes	10 (100.0)	5 (100.0)	5 (100.0)	
Contrast enhancement, *n* (%)				.44[a]
Yes	7 (70.0)	4 (80.0)	3 (60.0)	
No	1 (10.0)	1 (20.0)	0 (0.0)	

Unclear	2 (20.0)	0 (0.0)	2 (40.0)	
Necrosis, *n* (%)				1.00[d]
Yes	5 (50.0)	3 (60.0)	2 (40.0)	
No	5 (50.0)	2 (40.0)	3 (60.0)	
Hemorrhage, *n* (%)				
Yes	1 (10.0)	0 (0.0)	1 (20.0)	
No	7 (70.0)	5 (100.0)	2 (40.0)	.17[a]
Unclear	2 (20.0)	0 (0.0)	2 (40.0)	

Note. WT wildtype, pDMG pediatric diffuse midline glioma, C cervical vertebrae, T thoracic vertebrae, L lumbar vertebrae, IQR interquartile range, n.a. not applicable. Relative frequencies summing up to 100% per column.
[a] Fisher-Freeman-Halton test. [b] Mann-Whitney *U* test. [c] No statistics performed as values are identical in both groups. [d] Fisher's exact test of independence.
